# Utilization patterns of healthcare facility and estimated expenditure of PLHIV care under the Indonesian National Health Insurance Scheme in 2018

**DOI:** 10.1186/s12913-021-07434-9

**Published:** 2022-01-22

**Authors:** Ery Setiawan, Nurjannah Nurjannah, Kalsum Komaryani, Ryan Rachmad Nugraha, Hasbullah Thabrany, Farah Purwaningrum, Prih Sarnianto

**Affiliations:** 1 USAID Health Financing Activity, Central Jakarta, Indonesia; 2 Ministry of Health, South Jakarta, Indonesia; 3 Universiti Sains Malaysia, School of Social Sciences, Gelugor, Malaysia; 4 Pancasila University, South Jakarta, Indonesia

**Keywords:** HIV, JKN, Financing, PHLIV

## Abstract

**Background:**

This study analyzed current patterns of service use, referral, and expenditure regarding HIV care under the National Health Insurance Scheme (JKN) to identify opportunities to improve HIV treatment coverage. As of September 2020, an estimated 543,100 people in Indonesia were living with HIV, but only 352,670 (65%) were aware of their status, and only 139,585 (26%) were on treatment. Furthermore, only 27,917 (4.5%) viral load (VL) tests were performed. Indonesia seeks to broaden its HIV response. In doing so, it intends to replace declining donor-funding through better coverage of HIV/AIDS services by its JKN. Thus, this study aims to assess the current situation about HIV service coverage and expenditure under a domestic health-insurance funded scheme in Indonesia.

**Methods:**

This study employs a quantitative method by way of a cross-sectional approach. The 2018 JKN claims data, drawn from a 1% sample that JKN annually produces, were analyzed. Nine hundred forty-five HIV patients out of 1,971,744 members were identified in the data sample and their claims record data at primary care and hospital levels were analyzed. Using ICD (International Statistical Classification of Diseases and Related Health Problems), 10 codes (i.e., B20, B21, B22, B23, and B24) that fall within the categories of HIV-related disease. For each level, patterns of service utilization by patient-health status, discharge status, severity level, and total cost per claim were analyzed.

**Results:**

Most HIV patients (81%) who first seek care at the primary-care level are referred to hospitals. 72.5% of the HIV patients receive antiretroviral treatment (ART) through JKN; 22% at the primary care level; and 78% at hospitals. The referral rate from public primary-care facilities was almost double (45%) that of private providers (24%). The most common referral destination was higher-level hospitals: Class B 48%, and Class C 25%, followed by the lowest Class A at 3%. Because JKN pays hospitals for each inpatient admission, it was possible to estimate the cost of hospital care. Extrapolating the sample of hospital cases to the national level using the available weight score, it was estimated that JKN paid IDR 444 billion a year for HIV hospital services and a portion of capitation payment.

**Conclusion:**

There was an underrepresentation of PLHIV (People Living with HIV) who had been covered by JKN as 25% of the total PLHIV on ART were able to attain access through other schemes. This study finding is principally aligned with other local research findings regarding a portion of PLHIV access and the preferred delivery channel. Moreover, the issue behind the underutilization of National Health Insurance services in Indonesia among PLHIV is similar to what was experienced in Vietnam in 2015. The 2015 Vietnam study showed that negative perception, the experience of using social health insurance as well as inaccurate information, may lead to the underutilization problem (Vietnam-Administration-HIV/AIDSControl, Social health insurance and people living with HIV in Vietnam: an assessment of enrollment in and use of social health insurance for the care and treatment of people living with HIV, 2015). Furthermore, the current research finding shows that 99% of the total estimated HIV expenditure occurred at the hospital. This indicates a potential inefficiency in the service delivery scheme that needs to be decentralized to a primary-care facility.

## Background

Indonesia’s National Medium-term Development Plan (RPJMN) for 2020–2024 has mandated HIV care as one of its health priorities. Presidential Regulation Number 18 the Year 2020 regarding RPJMN states that one of the aims is to reduce the HIV rate to around 0.18 per 1000 population by 2024 [1]. Furthermore, in order to achieve the target, the Ministry of Health has produced an action-plan guideline with a set of intermediate outcomes, such as 90% PLHIV (People Living with HIV) recognizing their status, 90% of those PLHIV who are aware of their status are on ART, and 90% of those on ART are virally suppressed, in alignment with the Joint United Nations Program on HIV/AIDS targets in 2020. This will be expanded to triple 95% coverage when it comes to strategy by 2030.

**Fig. 1:**
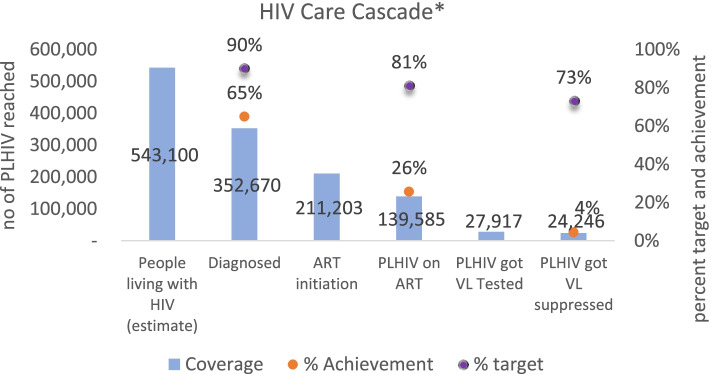
HIV National Cascade in Indonesia up to September 2020

Current estimates indicate that 543,100 people are living with HIV in Indonesia. As of September 2020, 65% (352,670) of people living with HIV knew their status, 26% (139,585) of people living with HIV were on ART, and 4% (24,246) of people living with HIV were virally suppressed (Fig. 1) [2]. Meanwhile, the global HIV target by 2030 is to have 95% case finding out of the total estimated HIV/AIDS patients; 90% PLHIV on ART; and 85% PLHIV on ART who virally suppressed [3] 2015). In contrast to the current situation, achieving the Joint United Nations Program on HIV/AIDS targets in 2030 will certainly require a concerted effort by the Government of Indonesia, together with other key stakeholders. However, the financing scheme for HIV care remains fragmented as many funds are being channeled in through different financing sources. There are three key sources of funding for the HIV program in Indonesia: (i) international (bilateral/multilateral) donor funding, (ii) public government (central and subnational, including JKN) funding and (iii) private funding. In line with the termination of external donor support, domestic funding needs to be leveraged mainly under the JKN scheme. Moreover, in an effort to bolster sustainable HIV financing through the JKN scheme, a general overview of the current utilization pattern is required as a policy baseline to increase treatment coverage.

JKN covers over 220 million members or about 83% of Indonesia’s entire population [4]. This integrated scheme is intended to ensure that an essential healthcare facility is accessible to members of JKN, regardless of their social-economic status or gender. Furthermore, it is based on the principle of social health insurance, as there is no screening on a pre-existing condition. Law Number 40 the Year 2004 regarding the National Social Security System in Article 22 stipulates that JKN benefits comprise a comprehensive package including preventive, promotive, curative, and rehabilitative services, which would encompass PHLIV services.

There are two kinds of payment systems applied under the JKN scheme that vary in terms of the level of the facility. Capitation payment is applicable for a primary-care facility while a Diagnosis Related Group (DRG) scheme (or Case-Based Group) is used at a hospital level. The difference between the payment systems is the rationale underpinning why CBG and capitation payment datasets warrant references in this study; the aim is to identify utilization patterns and estimated expenditure in primary care and hospitals.

A relatively recent evaluation from ILO in Indonesia concluded that five main parameters determine PLHIV access to JKN services, including issues due to not being covered in terms of membership in the JKN scheme, portability, tiered referrals, benefits packages, and treatment classes [5]. The study utilized a qualitative method and identified that 5% of PLHIV (out of the 258 PLHIV interviewed) have not accessed care from the JKN (National Health Insurance) scheme. This suggests that PLHIV has not used healthcare services. Moreover, the finding showed that among those 95% PLHIV who have accessed care through JKN, only 25% of them maintain their care using JKN contracted facilities.

Concerning the aforesaid national targets, it seems that ART coverage remains a key hurdle to address. It should be noted that ART coverage pertains more to the accessibility issue in HIV/AIDS. Regarding the national HIV/AIDS targets, a baseline assessment of the figure of the PLHIV service pattern is imperative. Having this kind of baseline assessment under the auspices of the National Health Insurance scheme is essential, as it represents population-based healthcare utilization [6, 7].

Intriguingly, the extent to which PLHIV maintains access to healthcare facilities in Indonesia, from a population-based perspective, is under-researched. In light of this, this study contributes empirically and scientifically by explaining the degree to which PLHIV maintains access to healthcare services, from a population-health perspective, by using claim data in hospitals and visit records in primary-care facilities under the JKN scheme. There are three objectives to this kind of assessment: (i) to ascertain the current HIV service patterns under the JKN; (ii) to identify areas of improvement so as to enable wider coverage, as well as improvement regarding the efficiency of care; (iii) to provide a basis to formulate further actions and policy for PHLIV in the JKN scheme.

In light of this background, this paper poses the following research question: what are the utilization patterns among PLHIV under the JKN scheme, and what is the estimated expenditure of those services? The first objective of this study is to understand the utilization patterns of PLHIV care through JKN, whereas the second objective is to estimate the total HIV-care expenditure, both in primary-care facilities and hospitals under the JKN scheme. The paper is structured as follows: the ensuing section discusses the methodology used in the study. The third section delves into findings regarding the themes of utilization patterns concerning HIV services in hospitals, the utilization pattern in primary care, the estimated total expenditure for HIV care, and patient access to the JKN scheme. The fourth section discusses these findings in the context of other HIV/AIDS-related studies. The final section summarizes the study and provides direction for future research.

## Method

### Analytical framework

This cross-sectional study employs secondary data analysis from JKN claim datasets to capture the utilization pattern of PLHIV in 2018 under the JKN scheme. There were four main areas of interest included in the study concerning the utilization of health services: 1) the portion of PLHIV services under the JK scheme; 2) service pattern at primary care facilities; 3) service pattern in hospitals; and 4) estimated HIV expenditure in the JKN scheme. To produce the figures for a baseline assessment, sequential data management processes (namely, duplication report, merging datasets, and data extrapolation using weight score) were performed.

### Data source

About data collection, this study utilized the 1% data sample of the JKN utilization datasets, both in primary care and hospital level in Indonesia. These datasets were obtained from BPJS Kesehatan - an administering body of JKN. The data were produced to give the public access to evidence, even though it may not be possible to access the full data, due to its size and consent. The weight score is applied to these datasets to convey how representative they are to the actual population size. In practice, there were four types of datasets included: 1) membership datasets; 2). primary-care datasets; 3) hospital datasets; 4) secondary diagnosis datasets. As already stated, 945 HIV patients were included in the analysis that represents health care consumption at the primary care level and hospitals with around 22 and 78% portion of service delivery, respectively.

The period of data included on these datasets covers utilization in 2018, as the most recent published dataset by BPJS Kesehatan. In terms of regional coverage, these datasets represented 34 provinces (all regions) in Indonesia. To extrapolate the data, the weight was applied to consider household and individual representativeness of each selected case or observation.

The data sets consisted of detailed information related to a patient’s characteristics, their primary diagnosis, secondary diagnosis, tertiary diagnosis, type of facility, level of services (outpatient or inpatient), level of severity, and a referral pattern from the primary-care facility to the hospital.

### Data management and analysis

The hospital dataset was combined with the secondary diagnosis datasets with an aim to select particular HIV cases in each level of care, as there was no secondary diagnosis variable in the original hospital dataset. By so doing, cases can be identified with an HIV-identifier code (ICD10 codes B20, B21, B22, B23, and B24) either in primary diagnosis, secondary diagnosis, or tertiary diagnosis, described as HIV-care observations. A similar process was also undertaken in primary care to identify HIV observation. Lastly, once both observations have been carried out, namely first patient ID was sorted, and the second duplication due to referral and repeat visits was excluded. Eventually, these data were combined into a membership dataset.

The economic burden of HIV care at the hospital level was estimated by organizing claim value per observation. This claim value was multiplied from an actual number of cases after considering observation weight. However, the estimation of economic burden at PHC was slightly different as a capitation scheme was applied for primary care services. Hence, the total economic burden at PHC was estimated by multiplying a portion of HIV care from the total service volume to the total annual capitation payment [8].

### Sample characteristics

The data sample characteristics covered patients included in the JKN data sample, who were 945 PLHIV enrolled in the health care facility during 2018 (Table 1). The number of male patients was slightly higher (50.95%) than female (49.05), and age distribution at around 2.33% for U5 children; 20.74% for children 6–18 years old (YO); 59.82% adult 18–55 YO; 17.11% for those more than 55 YO. The data shows that most patients were single, either unmarried (34.56%) or divorced (39.24%). In terms of a patient’s status in the family, most patients were the head of a household (36.69%) and children (37.78%). Healthcare utilization of HIV care was mostly accessed at region 1 (66.15%), which has a greater population and health facility distribution.

**Table 1:** Demographic characteristics of HIV patients claimed to JKN

Characteristic		% col
Gender	Male	50.95
	Female	49.05
Age group	Under Five	2.33
	Children (6–18)	20.74
	Adult (18–55)	59.82
	Elderly (> 55)	17.11
Marital Status	Not married	34.56
	Divorce	39.24
	Married	1.89
	Unidentified	24.31
Membership class	1st class	17.24
	2nd class	30.39
	3rd class	52.37
Family status	Head of HH	36.69
	Husband	2.04
	Spouse	19.33
	Children	37.78
	Additional dependent	4.16
Region	Region 1	66.15
	Region 2	5.18
	Region 3	12.41
	Region 4	2.89
	Region 5	13.37

### Strength and limitation

The strengths and limitations of this study were noted. First, the study was conducted in a large, integrated health system with access to samples of utilization datasets. This dataset includes any information regarding patient characteristics, and their health-seeking behavior either in a primary-care facility or at the hospital level, as well as clinical records by identifying the ICD 10 and ICD 9 CM. Another study by ILO/IAC examined the access pattern of patients to the JKN scheme – more importantly, patients with HIV and AIDS. However, this ILO/IAC study was limited to sampled respondents’ perceptions. Second, it used a national-level dataset representing a population perspective, even though there was limited access to only 1% data sample, with its weight for extrapolation perusal. Another strength of this paper is its scientific contribution, particularly if funding agencies are about to leave Indonesia, which will affect access to health services. That said**,** there are several limitations to this study, such as the inability to monitor treatment continuation or adherence to the guidelines, because the dataset did not cover information entailing when patients were diagnosed as HIV positive. Moreover, as the dataset covered only 2 years period without identifying the starting point of the observation, it may not be possible to apply a prospective cohort approach to this assessment. Therefore, it may not be possible to thoroughly describe the quality of services among PLHIV care under the JKN, including treatment adherence.

## Findings

There are four key themes in the research findings which correspond to utilization patterns among PLHIV under the JKN scheme and the estimated expenditure of these services. These themes – patient access to the JKN scheme, which is mostly through the hospital level; the utilization pattern for PLHIV through a public primary-care facility, the utilization pattern for HIV service delivery concentrated in public hospitals, and the total economic burden of HIV care under the JKN scheme – are all addressed in the ensuing paragraphs.

### Patient access to the JKN scheme

**Fig. 2:**
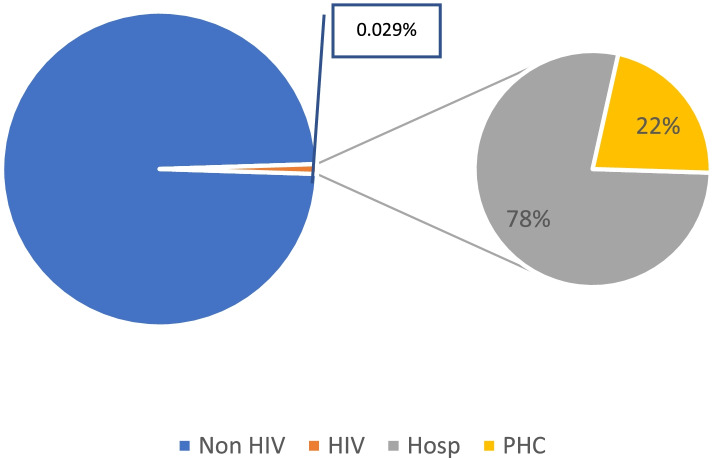
**a** Proportion of total PLHIV out of total JKN members. **b** Distribution of health care utilization -- level of providers

This pattern was estimated by comparing the patients to total JKN members ratio and the PLHIV on ART proportion to the total population. Nine hundred forty-five HIV patients out of 1,971,744 members were identified in the data sample and claim records at the primary care and hospital levels were analyzed. The proportion of PLHIV on ART to the total population was just around 0.040% while HIV patients’ access under the JKN scheme was around 0.029% of the total JKN members (Fig. 2a). It is, consequently, possible to infer that PLHIV access through the JKN scheme was estimated at around 72.5% among all PLHIV on ART.

In terms of delivery channels, most services were delivered at the hospital level (78%) and just around 22% were delivered at a primary-care facility (Fig. 2b). The MoH regulation No 21 the Year 2013 regarding the National HIV Program management initiated the HIV service delivery at the hospital as its capacity to test and to treat PLHIV [9]. Nonetheless, during recent years, the MoH has been strengthening the PHC capacity to test and treat PLHIV, so the utilization rate at PHC has been gradually increased as its capacity has improved.

### Utilization pattern in primary care

**Fig. 3:**
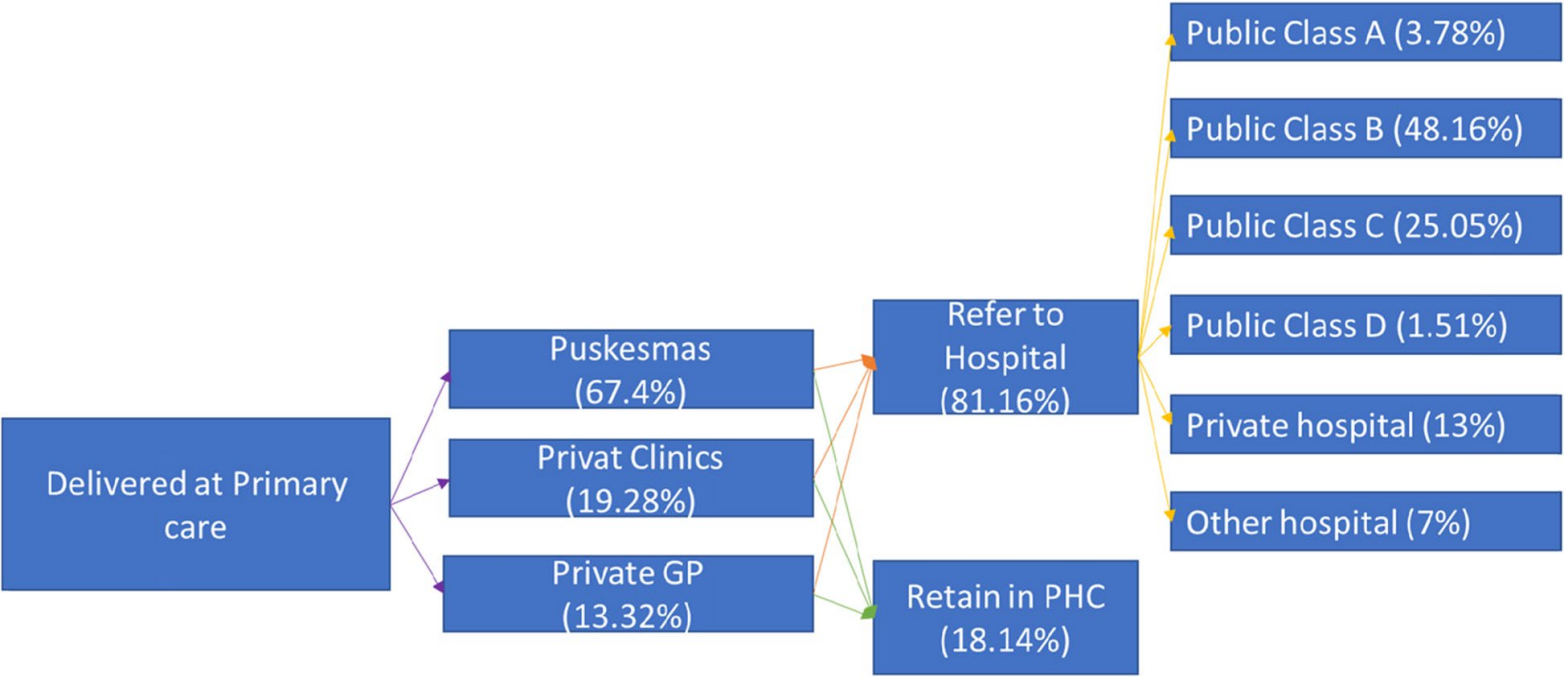
PLHIV utilization pattern in primary care facility

Public primary-care facilities remain at the forefront of service for PLHIV. Among 22% of HIV-care delivery at primary care facilities, most services were delivered at *puskesmas* or a public primary care facility (67.4%), while the remaining services were delivered at private facilities which are general practitioners and private clinics. This was consistent with the facility distribution, as *puskesmas* took around 43% of the total PHC contracted by BPJS Kesehatan. Moreover, most PHC trained for CST (care support treatment) were at *puskesmas* [2]. Among all service deliveries at PHC, most of them were referred to the hospital (81.16%) and around 18.14% remained at PHC. The most targeted referral hospitals were public hospitals Class B (48.16%), followed by Class C (25.05%) and private hospitals (13%), as represented by Fig. 3. Hospital is categorized as specified in class, D, B, C, and A, for both government and private-owned facilities based on certain parameters; i.e., specialty and infrastructure as specified in Government Regulation Number. Forty-seven the Year 2021 [10].

### Utilization pattern of HIV Service in Hospital

**Fig. 4:**
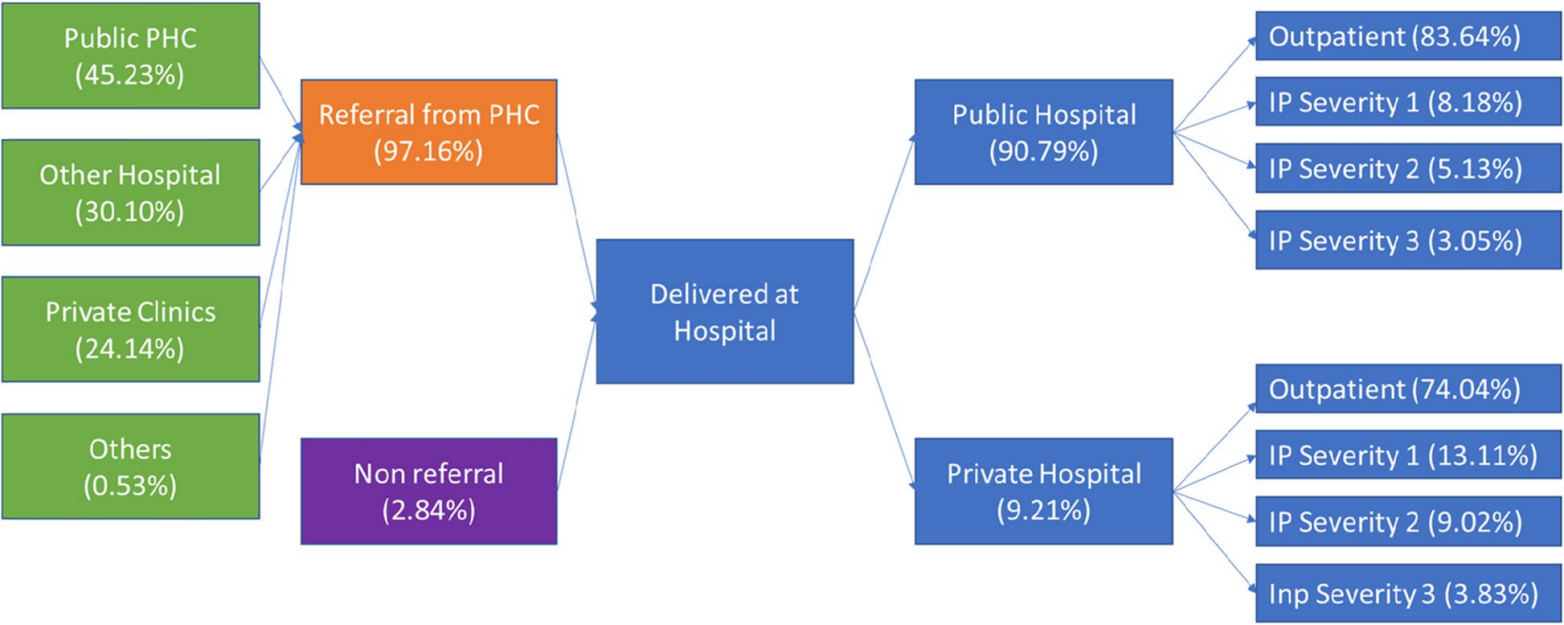
PLHIV utilization pattern in hospital

Data from JKN utilization demonstrates that most PLHIV care at the hospital comprised referrals at around 97.16% and approximately 2.84% of cases were admissions to the hospital without a referral (Fig. 4). Within all referral cases, most patients were referred from *puskesmas* (45.23%) and others to hospitals (30.1%). HIV-service delivery at the hospital was mainly centralized in public hospitals (90.79%) and around 9.21% was delivered in private hospitals. In terms of types of services, both kinds of hospitals have a similar pattern in that most cases are addressed at the outpatient department, at a rate of around 83.64% at public hospitals and 74.04% at private hospitals.

Analysis of the clinical aspect was performed by using ICD code as an identifier of observation. Interestingly, most cases were delivered at an outpatient department with no comorbidity and complication both in public and private hospitals. Furthermore, those cases with no comorbidity and complication were supposed to be delivered at a primary-care facility, which is more efficient in terms of cost and accessibility. However, there were no regulations nor clear guidelines for stable patients to be “down referred” (or transferred) to a primary-care facility as the current JKN scheme was limited to non-communicable diseases (NCD).

### Estimated Total expenditure of HIV care

The total economic burden of HIV care under the JKN scheme was estimated by incorporating the total claim at the hospital with an HIV identifier (ICD Code) and costs occurring at primary-care facilities were estimated as they were paid through a capitation scheme. The total claims at the hospital were calculated by multiplying cases by the weight before combining them to the claim value per observation. Estimating costs at PHC was indeed more challenging as it was paid by a capitation scheme which was not on a per-case basis. The PHC costs were estimated by using the HIV service proportion out of the total service delivery at the PHC level and then multiplied to the total capitation payment [11].

**Fig. 5:**
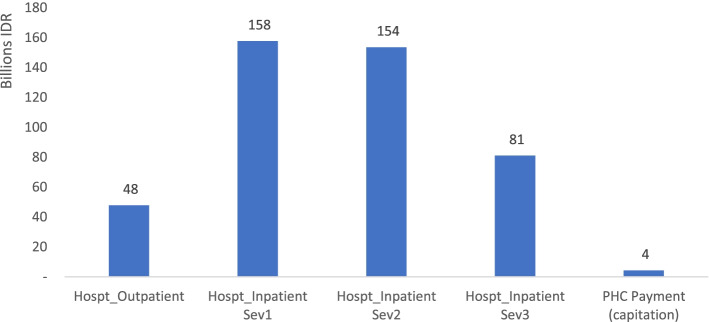
Total payment of HIV care under the JKN scheme. Notes: Sev - Severity; Hospt - Hospital; PHC - Primary Health Care

In general, the total economic burden of HIV care under the JKN scheme was around 444 billion IDR (around USD 30.5 Million), of which 99% occurred at the hospital level (Fig. 5).^1^ This finding was consistent with the NHA (National Health Account) figures in 2018, concluding that the HIV and STI (sexually transmitted infection) expenditure through the social health insurance scheme was around 624 billion IDR (around USD 42.8 Million), considering that a portion of HIV care was estimated at around 70% out of HIV-STI programs in Indonesia [12].

## Discussion

The study findings indicate that the role of the National Health Insurance (JKN) scheme was significant in order to cover PLHIV services, more importantly on ART. It was found that around 72.5% of PLHIV have accessed care through the JKN scheme, either in primary care or at the hospital level in 2018. This is inferred through an examination of patterns of health service utilization among PLHIV under the Indonesian National Health Insurance Scheme using a large, integrated dataset. A similar study was undertaken by HP+ study [13] in 2019 using the same data source, showing that PLHIV access to JKN for the period of 2016 was around 45%. The figure illustrates a significant improvement in around 2 years and this study complements the previous analysis [14].

There are, however, several hurdles with regard to accessing HIV testing services identified in earlier studies: first clients are failing to make time for testing; second is the issue of denying the notion of vulnerability to HIV; third is a feeling of consternation about losing the ability to be fully self-reliant; fourth is fear of an HIV diagnosis, which has been associated with stigma and discrimination; fifth is the fear of losing a position in the community due to societal norms of masculinity, which position men as physically strong (HIV being associated with weakness) [15–19]. Thus, considering that social determinants may affect health-seeking behavior, there will be a need for strategies through community-based services to be engaged.

An IAC study in 2019 focusing on all PLHIV involved within JKN membership shows that around 74% of those PLHIV have accessed care through JKN [5]. These findings align with this study’s assessment regarding the access rate of JKN services among PLHIV. The IAC study exemplifies a condition that among those who have accessed care through JKN, only 20% of those routinely visited a health facility using JKN [5]. There are several rationales for this relatively low percentage; namely, the complexity of a referral procedure, the length of waiting time for care, as well as stigma and discrimination as those PLHIV patients who gain access through JKN will be identified from the prior ICD code [20, 21]. However, the current analysis did not consider a continuation of treatment for one patient given the structure of the available datasets. Thus, as long as patients have received services through JKN, even once, they would be counted amongst the percentage with JKN access.

Alas, recent discussions from the Government of Indonesia’s viewpoint support a proposed framework to remove the HIV/AIDS services from the JKN benefit package, for several underlying reasons. First, HIV care is supposed to be fully covered by the Government of Indonesia, given its characteristics as a public good instead of under the JKN scheme. Second, the JKN scheme should be mandated to cover the personal health care [22]. In contrast, there are risks when HIV/AIDS services are removed from the JKN benefit package. One risk is that PLHIV is losing the opportunity to access the HIV/AIDS-related healthcare service that has reached around 72.5% coverage, currently, out of the total number of PLHIV on ART. Another risk is the increasing out-of-pocket costs that PLHIV may have to incur in accessing the HIV/AIDS-related healthcare service.

In addition, the integrated health financing scheme and service delivery mechanism through the JKN program will be much more efficient and offer higher quality due to care coordination, rather than having a more fragmented scheme using central government, local government, and an external donors’ channel. Importantly, the President of Indonesia has eventually decided to keep HIV care under the JKN scheme [23].

There is an underlying problem that should be addressed particularly to those who have not been covered by JKN or are inactive due to compliance to the contribution payment, as the IAC study showed that only 38% of patients routinely pay a JKN contribution. This study’s estimation found that the total expenditure for HIV care under the JKN scheme was 444 Billion IDR, which is around 0.1% out of the total health expenditure [12]. By considering the issue of contribution adherence among PLHIV, their health-seeking behavior cannot be neglected as combating HIV is one of the National Health Priority programs. Therefore, the Central Government needs to explore a policy option to cover all PLHIV under a type of scheme such as PBI (government paid membership), although this kind of scheme should be designed in a way to minimize stigma and protect the confidentiality of PLHIV. This study’s estimation shows that the increase of contribution paid by the Government for covering all PLHIV (352.000 people) was around 169 billion IDR or about 0.04% of the current total health expenditure.

Quintessentially, the study contributes in an empirical dimension to determine the PLHIV access pattern in health care facilities, either in primary care or a hospital at a macro level. The novelty of this study is that it is a population-based prospective analysis using JKN datasets: this has not been performed before in the context of Indonesia. More importantly, these findings will help the Ministry of Health, local governments, and policymakers in the health sector to formulate a better set of policy designs regarding the supply-side readiness, “down referral” mechanism, and PLHIV access to the JKN scheme.

## Conclusion

This study examined the access rate of PLHIV to the JKN (National Health Insurance) scheme, as well as their health-seeking behavior in both primary care facilities and hospitals. The analysis shows that around 72.5% of PLHIV on ART have accessed care through the JKN scheme: this was confirmed by the IAC study, which illustrated about 74% of PLHIV reached a health facility using the JKN scheme in 2019.

The study’s findings indicated that, in terms of the utilization pattern, the data shows that most services (78%) were delivered in hospitals while the rest were at a primary-care facility. Among those patients who were treated in primary-care facilities, approximately 81% were referred to hospitals for advanced treatment. In terms of health expenditure, an estimation of the total expenditure on HIV care under the JKN scheme was around 444 billion IDR, with 99% taking place at the hospital level. The higher claim at hospital emerged because of extremely high utilization at outpatient departments. This analysis found that approximately 83% of services at the hospital were delivered to outpatients and most of them had no opportunistic infection. What was given at the hospital, instead, was follow-up treatment. These kinds of cases are supposed to be treated at primary-care facilities to strengthen their role, including private clinics on this referral scheme and certainly for cost-containment purposes [22].

## Data Availability

The data that support the findings of this study are available from BPJS Kesehatan [Social Health Insurance Administrator] but restrictions apply to the availability of these data, which were used under license for the current study, and so are not publicly available. Data are however available from the authors upon reasonable request and with permission of BPJS Kesehatan. Further communication for data access can proceed through our corresponding author who is Ery Setiawan.

## Abbreviations

ART: Art Antiretroviral Therapy
AIDS: Acquired Immunodeficiency Syndrome
HIV: Human Immunodeficiency Virus
IAC: Indonesian AIDS Coalition
ILO: Human Immunodeficiency Virus
JKN: *Jaminan Kesehatan Nasional* (National Health Insurance)
PLHIV: People Living with HIV
RPJMN: *Rencana Pembangunan Jangka Menengah Nasional* (National Development Mid-Term Plan)
VL: Viral Load

## Footnotes

1 Currency conversion refers to Bank of Indonesia rate on 31st December 2018, as around IDR 14.553 per USD
